# Uterine Polypi and Febroids

**Published:** 1879-05

**Authors:** 


Uterine Polypi and Fibroids.—I saw him operate on a woman for the removal of a uterine fibroid polypus that had descended into the vagina and filled this organ so completely that the evacuation of both the rectum and bladder was completely obstructed.

The growth was so large that he was unable to pass the écraseur over the entire mass to its cervical attachment, and hence had to pass the chain of the instrument around its segment, and remove it in three separate portions.

The last was so large that he had to extract it with a pair of obstetrical forceps. He said: "Whether these growths be solid, soft or cystic, there are only three modes of removing them that I recognize as proper to adopt, and these are, the knife or scissors, the écraseur and the galvano-cautery. When they are attached high up in the uterus, I do not like the galvano-cautery, for the reason that you can never be really certain what you are burning, and you may destroy tissues that will lead to disastrous consequences."

The use of ligature for the removal of these foreign bodies he especially condemned. In the early years of his professional life, he had seen two patients die of septicæmia following the removal of a uterine polypus in this manner.

We must remember, in all our operations upon the uterus, that although we have to do with an organ that will tolerate a good deal of mechanical violence, that we have also to do with a mucous surface that will the most rapidly absorb effete matter of any tissue in the entire body; and when a polypus is ligated it soon becomes a putrid mass, from which the whole economy may become rapidly contaminated. The twisting off of polypi he also considers a bad practice, and has seen at least one case where the extensive laceration of the mucous surface of the womb attending the operation resulted in a metritis from which the patient died.

Prof. Schröder has in his wards at the Charité this winter some interesting cases to illustrate his management of different forms of uterine fibroids. In our treatment of these cases, he says, we should remember one fundamental rule, which is, that in the history of every such case there usually comes a time when the morbid growth will cease to increase in size, begin perhaps to undergo a retrograde metamorphosis, and become in time entirely innoxious as far as the well-being of our patient is concerned. Directly opposite to this are the facts connected with the history of most cases of ovarian cysts. Their tendency is ever to increase in size, and their removal will sooner or later be imperatively demanded. Keeping these facts in view, we seize upon the most appropriate time for our operative interference. In the management of a case of uterine fibroid, he says, investigate your case accurately as to this fact. Is its attachment situated upon the lining membrane of the *body* of the womb, or upon the *cervix* uteri. If it has its origin from the body of the womb, you are to consider it as a *noli me tangere*, unless it be the direct cause of symptoms that are likely to prove dangerous to the life of the woman. On the other hand, if it spring from the cervix uteri, you may operate on it in almost any way with comparative safety.

To arrest the hæmorrhages that accompany these cases, he swabs out the inner surface of the womb with either the tincture of iodine or a solution of one of the astringent salts of iron.

A question in which I have been greatly interested, and upon which I have interviewed everybody, is the value of the hypodermic injection of ergotine in the treatment of uterine fibroid.

Prof. Schröder says that he has often seen cases greatly benefitted by this treatment, but has never seen a case entirely cured by it. By its use the hæmorrhage will often cease, and the tumor become greatly lessened in size. To test the remedy he says you must use at least one hundred injections. He makes them into the cellular tissue of the abdominal walls, and repeats them as often as every alternate day. As so protracted a use of an agent that is often very painful, taxes to the utmost the patience of both physician and patient, few carry it out thoroughly.

Braun, of Vienna, makes these injections into the outer aspect of the thigh, where they are better borne. He uses also Bourbellon's ergotine, and none other, as he says you never have an abscess follow its use. Its name, I believe, is derived from a Swiss chemist who manufactures it.

Dr. Routh, of Dorset House Hospital for Women and Children, in London is the greatest enthusiast of any I have met in Europe as regards the efficacy of his treatment of intra-uterine fibroids. His plan is to first puncture the tumor by passing a sharp pointed instrument into it about the size of a number six English catheter. The depth to which he makes this puncture will depend upon the size of tumor, but will usually be to about one half the thickness of the growth. After making his puncture, he introduces into the hole thus made a wire of nearly the same size, heated to a red heat. He claims that in this way you can excite an inflammatory change in the body of the fibroid, that will lead to its absorption, and that too without any dangers of a septic process following the procedure, which would be likely to occur if you attempted to accomplish the same object in any other way. He says that he has never failed to benefit a case that he has treated in this manner.

He was surprised when I told him that I had seen Prof. Péan, of Paris, carry out the same idea in the management of some cases at the St. Louis.

